# Erosion rate study at the Allchar deposit (Macedonia) based on radioactive and stable cosmogenic nuclides (^26^ Al, ^36^ Cl, ^3^ He, and ^21^ Ne)

**DOI:** 10.1002/2015GC006054

**Published:** 2016-02-14

**Authors:** M. K. Pavićević, V. Cvetković, S. Niedermann, V. Pejović, G. Amthauer, B. Boev, F. Bosch, I. Aničin, W. F. Henning

**Affiliations:** ^1^ Chemistry and Physics of MaterialsUniversity of SalzburgSalzburgAustria; ^2^ Faculty of Mining and GeologyUniversity of BelgradeBelgradeSerbia; ^3^ Helmholtz‐Zentrum Potsdam—Deutsches GeoForschungsZentrum GFZPotsdamGermany; ^4^ Faculty of PhysicsUniversity of BelgradeBelgradeSerbia; ^5^ Faculty of Mining and GeologyUniversity of ŠtipŠtipMacedonia (FYROM); ^6^ Gesellschaft für Schwerionenforschung GSIDarmstadtGermany; ^7^ Argonne National Laboratory, Physics DivisionArgonneIllinoisUSA

**Keywords:** erosion, cosmogenic nuclides, Allchar, paleo‐depth, pp‐neutrinos, 205Tl

## Abstract

This paper focuses on constraining the erosion rate in the area of the Allchar Sb‐As‐Tl‐Au deposit (Macedonia). It contains the largest known reserves of lorandite (TlAsS_2_), which is essential for the LORanditeEXperiment (LOREX), aimed at determining the long‐term solar neutrino flux. Because the erosion history of the Allchar area is crucial for the success of LOREX, we applied terrestrial in situ cosmogenic nuclides including both radioactive (^26^Al and ^36^Cl) and stable (^3^He and ^21^Ne) nuclides in quartz, dolomite/calcite, sanidine, and diopside. The obtained results suggest that there is accordance in the values obtained by applying ^26^Al, ^36^Cl, and ^21^Ne for around 85% of the entire sample collection, with resulting erosion rates varying from several tens of m/Ma to ∼165 m/Ma. The samples from four locations (L‐8 CD, L1b/R, L1c/R, and L‐4/ADR) give erosion rates between 300 and 400 m/Ma. Although these localities reveal remarkably higher values, which may be explained by burial events that occurred in part of Allchar, the erosion rate estimates mostly in the range between 50 and 100 m/Ma. This range further enables us to estimate the vertical erosion rate values for the two main ore bodies Crven Dol and Centralni Deo. We also estimate that the lower and upper limits of average paleo‐depths for the ore body Centralni Deo from 4.3 Ma to the present are 250–290 and 750–790 m, respectively, whereas the upper limit of paleo‐depth for the ore body Crven Dol over the same geological age is 860 m. The estimated paleo‐depth values allow estimating the relative contributions of ^205^Pb derived from pp‐neutrino and fast cosmic‐ray muons, respectively, which is an important prerequisite for the LOREX experiment.

## Key Points

- Erosion rate of a unique Tl‐deposit is studied by radioactive/stable nuclides
- The vertical erosion rate is obtained for the locations with most Tl‐rich ore
- The study contributes to determining the long‐term average flux of solar pp‐neutrinos

## 1 Introduction

### 1.1 The LOREX Project

The Allchar Sb‐As‐Tl‐Au hydrothermal deposit is related to a Pliocene volcano‐intrusive complex located at the north‐western margins of the Kožuf Mountains and close to the border between the Former Yugoslav Republic of Macedonia and Greece [*Frantz et al*., 1994; *Janković and Jelenković*, 1994]. Allchar is a world‐class thallium deposit unique by its quantity of thallium reserves and by the number of thallium minerals discovered. It contains sufficient quantities of lorandite (TlAsS_2_), and because this mineral can be used as a geochemical detector of proton‐proton (pp) solar neutrinos [*Freedman et al*., 1976], the deposit has gained the interest of a wider community of astrophysicists, nuclear physicists, and geochemists [e.g., *Frantz et al*., 1994].

The main goal of the LOREX (LORanditeEXperiment) Project is to determine the average fluence of solar pp‐neutrinos Φ_ν_ for the age period of the lorandite in the Allchar deposit [*Pavićević*, 1994], according to the following equation:

(1) $$\Phi_{\nu }\sigma_{\nu }=C\frac{{\left(N^{205}Pb\right)}_{\text{exp}}-{\left(N^{205}Pb\right)}_{B}}{m(1-e^{\lambda T})}$$

where C is a constant (C = 3.79 × 10^−19^ mol a^*−*^^1^), σ_ν_ is the cross section for the capture of solar (pp) neutrinos by ^205^Tl, (N^205^Pb)_exp_ is the experimentally determined number of ^205^Pb atoms in lorandite of mass *m*, (N^205^Pb)_B_ is the number of ^205^Pb atoms in lorandite resulting from background reactions, λ is the decay constant of ^205^Pb (λ = 4.68 × 10^*−*^^8^ a^*−*^^1^), and T = 4.3 Ma is the age of the thallium mineralization in Allchar [*Neubauer et al*., 2009].

The number of ^205^Pb atoms (N^205^Pb)_B_ (see equation (1)) produced by cosmic radiation, mostly by (μ,pn)‐reactions, and by natural nuclear reactions connected to U and Th decay, must be determined quantitatively. In this context, the knowledge of the erosion rate of the overburden rocks since the formation of lorandite, i.e., the paleo‐depth as a function of time, is of utmost importance [*Pavićević*, 1994]. This is why at the beginning of LOREX, by the end of the eighties and the beginning of the nineties, we started with detailed studies of the erosion rate in Allchar. These studies were twofold, focusing on geomorphological analysis and on applying terrestrial in situ cosmogenic nuclides (hereafter TCN). The first TCN studies based on ^36^Cl [*Dockhorn et al*., 1991] suggested a small erosion rate in this area, and therefore a high contribution of cosmic‐ray‐produced ^205^Pb, which would make further investigations on the LOREX project pointless. However, the subsequent measurements of ^26^Al concentrations suggested higher erosion rates [*Pavićević et al*., 2004], what encouraged further research.

The aim of this paper is to provide an estimate of the erosion rate in the area of the Allchar deposit by applying both radioactive (^26^Al and ^36^Cl) and stable (^3^He and ^21^Ne) cosmogenic nuclides in various minerals (quartz, dolomite/calcite, sanidine, and diopside). Although the obtained values may not reflect precise erosion rates in the studied region, they should provide a best approximation, i.e., an order of magnitude estimate of the prevailing erosion rate. Such estimates are sufficient for assessing the order of magnitude of the contribution of cosmic radiation in the ^205^Pb production during the last 4.3 Ma and thereby represent an important step forward in demonstrating the feasibility of the LOREX Project. This is because, before continuing with time‐consuming and expensive analytical procedures, we need to know the order of magnitude of the denudation rate.

### 1.2 Geological Outline of the Allchar Area

The Allchar deposit is spatially and petrogenetically related to a Pliocene volcano‐intrusive complex, which is situated between the Pelagonian tectonic unit and the Vardar zone suture in the east and west, respectively [e.g., *Karamata*, 2006; *Schmid et al*., 2008]. The immediate volcanic basement consists of Triassic‐Jurassic rock series, predominantly dolomite and dolomitic marble, limestone, sandstone, clayey schist, quartzite, and chert. Detailed information about the geology of Allchar can be found in *Boev* [1988], *Frantz et al*. [1994], *Janković and Jelenković* [1994], *Karamata et al*. [1994], and *Amthauer et al*. [2012], among others.

Three phases of magmatic activity are distinguished in the Allchar area. The first one took place in the Miocene (14.3–8.2 Ma), when numerous calc‐alkaline dykes were emplaced. The second phase occurred in the Pliocene, producing latite, quartzlatite, and trachyte to subordinate andesite/dacite subvolcanic intrusions. The third phase occurred between 6.5 and 1.7 Ma [*Boev*, 1988], and during this event the thallium mineralization also formed. The andesite lavas and tuffs of the area Crven Dol formed 6.5–3.9 Ma, the tuffs from the localities Vitačevo and Rudina formed from 5.1 ± 0.1 to 4.31 ± 0.02 Ma, whereas the Kojčov Rid latites originated between 4.8 and 3.3 Ma [*Neubauer et al*., 2009]. *Troesch and Frantz* [1992] determined the geological age of a sanidine from adit Nr. 21 (Crven Dol) to be 4.22 ± 0.07 Ma, which is in very good agreement with the age of the sanidine from Rudina, i.e., 4.31 ± 0.02 Ma [*Neubauer et al*., 2009]. The Allchar volcanic rocks are overlain by volcano‐sedimentary rocks mainly represented by tuffaceous dolomite that was deposited in sublacustrine basins.

The Allchar Sb‐As‐Tl‐Au deposit is a NNW‐SSE stretching, 2 km long and 300–500 m wide zone which comprises several ore bodies. The mineralization is mainly localized along unconformities between the basement rocks and the volcanic or volcaniclastic cover. The precipitation of the Sb‐As‐Tl‐Au mineralization was dated by the Ar/Ar method at 4.31 ± 0.02 Ma [*Neubauer et al*., 2009]. It was accompanied by intensive hydrothermal alteration predominantly represented by silicification and argilitization. The main thallium ore bodies are Crven Dol and Centralni Deo, and they are situated along the contacts between subvolcanic andesites and their volcaniclastic counterparts and hydrothermally altered dolomites [*Boev and Jelenković*, 2012].

There are no accurate data about active uplift in this part of the Balkan Peninsula. However, it is documented that from the late Miocene onward, this area underwent mainly strike‐slip tectonics [*Dumurdzanov et al*., 2005; *Hoffmann et al*., 2010].

Paleoclimatic reconstructions and the timing of glacial retreat in the Allchar area are poorly constrained. Namely, the data about these events are either very limited and lack quantitative measurements [*Cvijić*, 1903, 1913], or they mostly refer to broad regional scales, sometimes encompassing almost the entire Balkan Peninsula [*Menković et al*., 2004]. Nevertheless, the existing studies suggest that although a significant part of the Balkan area was covered by ice during the Quaternary, it is very likely that the Last Glacial Maximum affected only the highest altitudes.

## 2 Sampling and Experimental Methods

### 2.1 Locations of Samples

**Figure 1:**
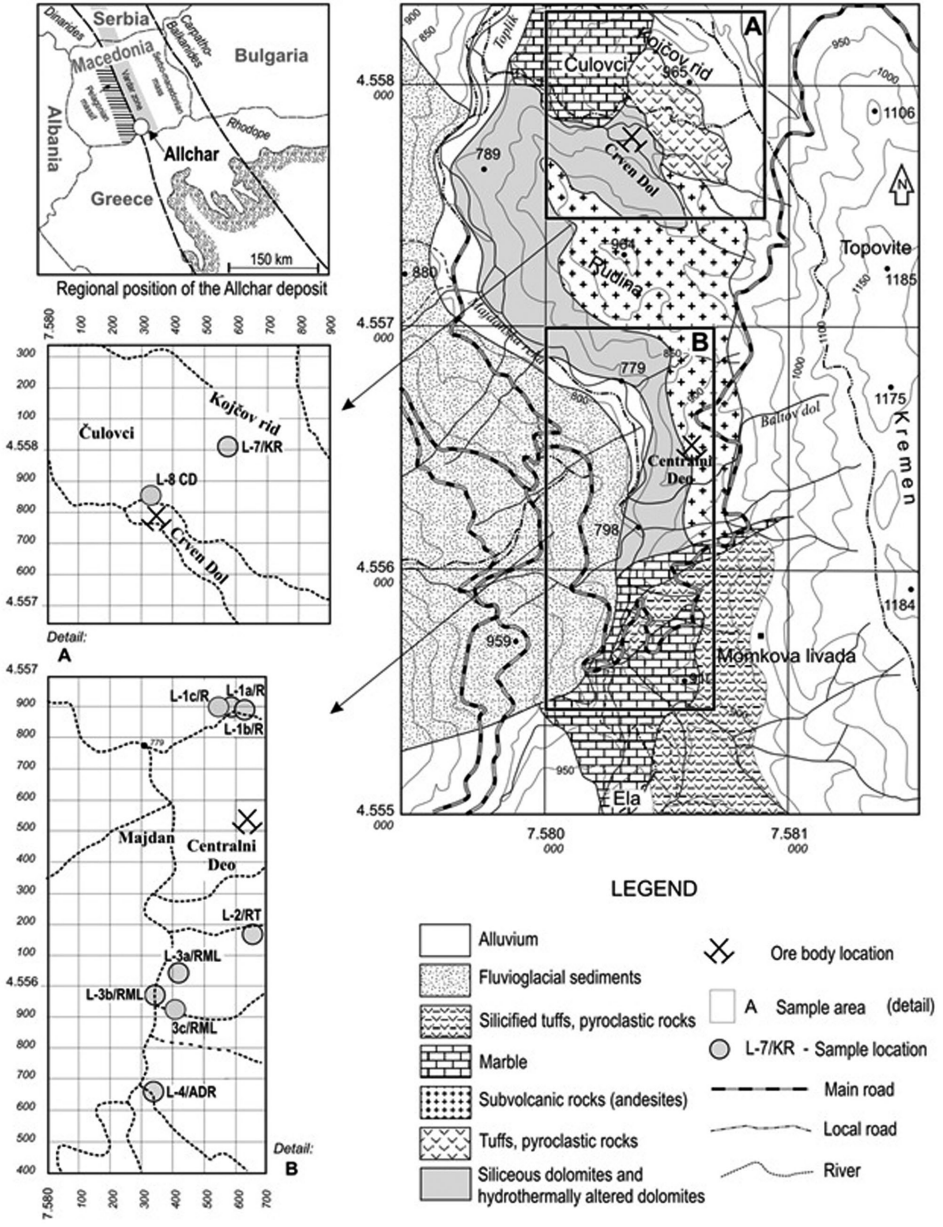
*Right*: A simplified geological map of the wider area of the Allchar deposit (modified from *Janković* and *Jelenković* [1994]). *Detail A*: Map of the sampling locations at the Crven Dol locality. *Detail B*: Sampling locations at the Centralni Deo locality. *Gray circles*: Positions of samples collected for analysis of cosmogenic nuclides.

The Allchar area is mostly covered by vegetation, therefore only a few locations were suitable for sampling. All samples were ranging in weight from 2 to 5 kg and were taken from the surface to a depth of 10 cm, except for three calcite/dolomite samples from depths of 1.3 m (Do 50L/13), 2.5 m (Do 1/13), and 5 m (Do 8/13).Over more than 10 years, we have been collecting samples that would primarily contain quartz that is commonly used for studies of the cosmogenic radionuclides ^10^Be and ^26^Al. Figure 1 shows a map of 11 sampling locations, which are either near or above the lorandite ore bodies of Crven Dol and Centralni Deo. This ensures that the erosion rate estimates are significant for the determination of the paleo‐depth of the thallium mineralization.

All the samples were studied petrologically and mineralogically, by applying optical microscopy, X‐ray diffraction, and, in some cases, Scanning Electron Microscopy and Energy Dispersive X‐ray Spectroscopy (SEM‐EDX). Information about the sampling locations and the petrography of samples is summarized in Table 1.

**Table 1:** Rocks, Minerals, and Site Parameters for the Sampling Location Sites in Allchar

Location	Sample Number	Petrography and Mineral Composition	Latitude	Longitude	Elevation (m)	Slope (°)
L‐7/KR	Di/09	Slightly altered volcaniclastic rock; Plagioclase, biotite, quartz, christobalite, sanidine, diopside, albite	41°10′5″	21°57′34.3″	965	5
L‐8/CD	Do 8/13	Fine‐grained dolomitic limestone with stylolitic textures	41°9′33.4″	21°57′31″	821	20
L‐1a/R	Sa/08	Hydrothermally altered dolomite; Hydrothermal quartz, pyrite, marcasite, sanidine, Fe‐hydroxides, jarosite, K‐feldspar	41°09′14.2″	21°57′34.8″	855	35
L‐1b/R	4b/2	Hydrothermally altered and mineralized volcaniclastic rock with abundant quartz veins Hydrothermal quartz, crystals about 5–15 mm	41°09′13.7″	21°57′36.2″	860	35
L‐1c/R	Do 50L/13	Hydrothermally altered and mineralized volcaniclastic rock containing quartz veins	41°09′14.35″	21°57′34″	850	45
L‐2/RT	2.1	Hydrothermally altered and mineralized volcaniclastic rock; the sample 2.1 represents a composite of three rock samples slightly differing in mineralogy: (a) hydrothermal quartz, scorodite, realgar, barite, Fe‐hydroxide, (b) hydrothermal quartz, realgar, orpiment, scorodite, sericite, Fe‐hydroxides, and (c) hydrothermal quartz from a vein with quartz crystals about 10–20 mm in diameter	41°08′40.5″	21°57′37″	895	45
L‐3a/RML	3.2/2 6 6/2‐1 6/2‐2 6/9	All the samples represent mineralized and hydrothermally altered volcaniclastic rocks with similar mineralogy Hydrothermal quartz, sericite, jarosite and Fe‐hydroxide	41°08′34.7″	21°57′29.4″	850	20
L‐3b/RML	3.1/2 5 5/9	The same as above	41°08′31.4″	21°57′27″	830	25
L‐3c/RML	Do 1/13	The same as above	41°08′28.6″	21°57′30″	845	35
L‐4/ADR	1.3/2 2.1/9	The site is represented by silicified and mineralized volcaniclastic rock Sample 1.3/2 is a mixture of hydrothermal quartz, sericite, marcasite, barite, jarosite, Fe‐hydroxide Sample 2.1/9is a vein composed of hydrothermal quartz with crystals about 5–8 mm in diameter	41°08′17.4″	21°57′27″	820	15

### 2.2 Mineral Separation Procedures

#### 2.2.1 Preparation of Quartz Concentrates

Total quartz concentrates (60–70% pure silica) were produced from pristine rock samples (about 3–5 kg each), applying standard mineral separation techniques [*Pavićević et al*., 2006]: (I) crushing and grinding, (II) sieving to selected grain sizes <0.5 mm, and (III) separation of quartz concentrates on the shaking table with water. From the quartz concentrates, two samples of 80–100 g each were taken and were etched following the procedure described in *Pavićević et al*. [2004]. The etching was performed repeatedly (3–5 times) with 30% H_2_SiF_6_ and “aqua regia” (HCl:HNO_3_ = 3:1) at room temperature over a time period of 3–4 days, under constant shaking. After each step, the sample was washed with bidistilled water and dried at 80°C, along with a control of weight loss. The quality of each treatment was checked with X‐ray diffraction. At the end of the treatment, we obtained pure stoichiometric quartz with >99% SiO_2_ [*Pavićević et al*., 2006].

The preparation procedure for hydrothermal quartz was similar to the one applied for total stoichiometric quartz. Relatively big (∼5–15 mm) crystals of hydrothermal quartz were sampled at three locations (cf. Tables 4 and 5). 2–3 g of crystals of hydrothermal quartz were etched with 30% H_2_SiF_6_ and “aqua regia” (HCl:HNO_3_ = 3:1). The quality of each treatment was checked with X‐ray diffraction and SEM‐EDX techniques.

#### 2.2.2 Preparation of Calcite/Dolomite Concentrates

Samples of calcite/dolomite were taken at the localities L‐1c/R, L‐3c/RML, and L‐8/CD (sample numbers: Do 50L/13, Do 1/13, and Do 8/13) in quantities from 3 to 4 kg (Table 1). For producing calcite/dolomite concentrates, a standard procedure of mineral separation was carried out. It involved crushing, sieving, and grinding to a fraction of <0.5 mm, then, separation on a shaking table and magnetic separation for reducing quartz concentrations. Because they contain both minerals, we labeled these samples calcite/dolomite concentrates.

#### 2.2.3 Preparation of Sanidine and Diopside

Sanidine (K_0.75_Na_0.25_AlSi_3_O_8_) was separated from a hydrothermally altered and mineralized volcaniclastic rock (3–4 kg of a sample from the locality L‐1a/R; Table 1). The classical procedure comprised grinding, crushing, and sieving (<125 µm to <2 mm), and the obtained fraction 400–630 µm was used for further treatment. Pure sanidine crystals (∼500 mg) were separated by hand‐picking using a binocular microscope. X‐ray diffraction data (performed in duplicate) confirmed a sanidine crystal structure.

A similar procedure was applied for diopside (MgCaSi_2_O_6_) separation. One sample of 2 kg from the locality L‐7/KR (Table 1) underwent grinding, crushing, and sieving (<125 to <355 µm). The fraction 250–355 µm was further separated using a shaking table. After that, pure diopside crystals (∼370 mg) were separated using magnetic separation and hand‐picking. X‐ray diffraction and SEM‐EDX analyses confirmed that the obtained separates consisted of >96% pure diopside.

### 2.3 Determination of Cosmogenic Nuclide Concentrations

#### 2.3.1 Measurements of ^26^Al in Total Stoichiometric Quartz

Fifteen samples of total stoichiometric quartz in quantities of ca. 20 g/sample were sent in two series to the PRIME Lab (Purdue Rare Isotope Measurement Laboratory, Purdue University) for Acceleration Mass Spectrometry measurements of ^26^Al. Each sample was dissolved in 5:1 HF/HNO_3_ and after dissolution (no residue left) an aliquot was taken for stable ^27^Al determination by Inductively Coupled Plasma‐Optical Emission Spectrometry. To the rest of the samples, the standard chemical procedures used at PRIME lab were applied, finally yielding targets in the form of Al_2_O_3_.For ^26^Al, the standard of *Nishiizumi* [2004] was used, which corresponds to a ^26^Al half‐life of 708 ± 17 ka.

#### 2.3.2 Determination of ^36^Cl in Calcite/Dolomite

The mineral composition of calcite/dolomite concentrates was characterized both qualitatively and quantitatively by X‐ray powder diffraction including a Rietveld analysis. This investigation revealed that the samples were predominantly represented by calcite (95.8–99.2%), whereas dolomite was subordinate (0.5–3.4%). The concentration of Ca and K contents was determined by Inductively Coupled Plasma Mass Spectrometry (ICP‐MS). This showed that K concentrations were very low (ca. 0.01%), indicating that ^36^Cl production from K is negligible. Similarly, the measured values of stable chlorine are low (mostly <0.5 ppm). This suggests that the contribution of ^36^Cl from thermal neutron capture of ^35^Cl is only about 0.1% of that produced from Ca [*Phillips et al*., 2001], and can therefore be neglected as well.

The concentrates of calcite/dolomite samples (ca. 150 g each) were sent to the PRIME Lab, where the final sample preparation and the AMS measurements of ^36^Cl were carried out.

#### 2.3.3 Determinations of Cosmogenic ^3^He and ^21^Ne in Diopside, Quartz, and Sanidine

Cosmogenic ^21^Ne concentrations were determined at GFZ Potsdam in one sanidine and six quartz separates, whereas the cosmogenic ^3^He concentration was assessed in one diopside separate. The samples were wrapped in Al foil and loaded in the sample carrousel above the extraction furnace, which was then baked at 100°C for about one week. The noble gases were extracted by stepwise heating (400, 600, 800, and 1200°C for quartz; 600, 800, and 1750°C for sanidine; 900 and 1750°C for diopside) for at least 20 min each. Chemically active gases were removed by two Ti sponge getters and two SAES (ZrAl) getters, and He, Ne, and Ar‐Kr‐Xe were separated from each other by trapping in a cryogenic adsorber and subsequent sequential release. Noble gas concentrations and isotopic compositions were determined in a VG5400 sector field mass spectrometer. Results were corrected for isobaric interferences, instrumental mass fractionation, and analytical blanks. Further details about the analytical procedures can be found in *Niedermann et al*. [1997].

## 3 Results

### 3.1 Cosmogenic and Stable Nuclides

The results of ^26^Al measurements of 15 samples are shown in Table 2, those of ^36^Cl measurements are reported in Table 3. Stable Al concentrations in quartz from Allchar are uncommonly high, ranging from 2520 to 3014 µg/g [*Pavićević et al*., 2006]. This caused a decrease in the precision of ^26^Al/^27^Al measurements, which have a detection limit of ∼10^−15^ for a stable Al concentration range of 100–200 µg/g.

**Table 2:** Acceleration Mass Spectrometry Measurements of ^26^Al in Quartz

	Sample Name	Sample Mass	Native Aluminum	^27^Al Carrier	^26^Al/^27^Al	^26^Al/^27^Al StdDev	^26^Al	^26^Al StdDev	^26^Al StdDev
Location		(g)	(mg)	(mg)	(× 10^−15^)	(× 10^−15^)	(10^5^ at/g SiO_2_)	(10^5^ at/g SiO_2_)	(%)
L‐1a/R	4a/2^a^	19.8075	1.51		0	180	0		
L‐1b/R	4b/2	20.0555	1.30		83	72	1.2	1.0	87
L‐2/RT	2.1^a^	19.9885	56.99		0	0.8	0		
L‐3a/RML	3.2/2	20.0278	40.97		13.8	6.8	6.3	3.1	49
L‐3a/RML	3.2/2^b^	24.697	50.0	1.037	17.9	6.3	8.2	2.9	35
L‐3a/RML	6	19.9119	58.93		10.3	3.7	6.8	2.5	36
L‐3a/RML	6/2	20.1449	40.25		19.6	5.1	8.8	2.3	26
L‐3a/RML	6/2^b^	24.671	53.6		11.4	3.4	5.5	1.7	30
L‐3a/RML	6/9	19.9863	58.47		8.4	3.3	6.7	1.2	20
L‐3b/RML	3.1/2	19.8943	45.43		8.5	2.7	4.3	1.4	32
L‐3b/RML	3.1/2^b^	24.769	57.3	1.116	12.6	4.2	6.7	2.2	33
L‐3b/RML	5	19.8894	59.94		6.2	3.6	4.2	2.4	58
L‐3b/RML	5/9	19.8293	59.30		7.0	2.8	4.8	0.8	17
L‐4/ADR	1.3/2^a^	20.0776	59.00		0	1.4	0		
L‐4/ADR	2.1/9	19.8519	54.42		2.0	1.8	1.3	1.1	87

a ^26^Al values below detection limit.

b Duplicate measurements.

**Table 3:** Acceleration Mass Spectrometry Measurements of ^36^Cl

	Sample Name	Sample Mass	Total Chloride in Rock	Total Chloride in rockStdDev	^36^Cl/Cl^a^	^36^Cl/Cl StdDev	^36^Cl	^36^Cl StdDev
Location		(g)	(ppm)	(ppm)	(× 10^−15^)	(× 10^−15^)	(10^4^ at/g of the Sample)	(10^4^ at/g of the Sample)
L‐1c/R	Do 50L/13	14.6023	20.8	0.6	34.0	1.4	3.70	0.31
L‐3c/RML	Do 1/13	13.1609	bdl^b^		54.3	2.7	5.23	0.44
L‐8/CD	Do 8/13	14.7459	bdl^b^		32.3	1.6	2.16	0.30

a Cl in denominator is total Cl (Cl in the rock + Cl added by dilution spike).

b bdl—below detection limit.

The same sample set was analyzed for ^10^Be as well. However, compared to both ^26^Al and ^21^Ne, the ^10^Be concentrations were apparently an order of magnitude higher, and consequently the nominal erosion rates calculated on the basis of these ^10^Be AMS measurements were considerably smaller than those obtained on the basis of ^21^Ne, ^26^Al, and ^36^Cl concentrations. Because, in particular, a ^10^Be/^21^Ne ratio higher than the production ratio is physically impossible, such discrepancy suggests that our quartz purification procedure did not successfully remove all meteoric ^10^Be from the grain surfaces, and therefore the ^10^Be results are not reported here.

**Figure 2:**
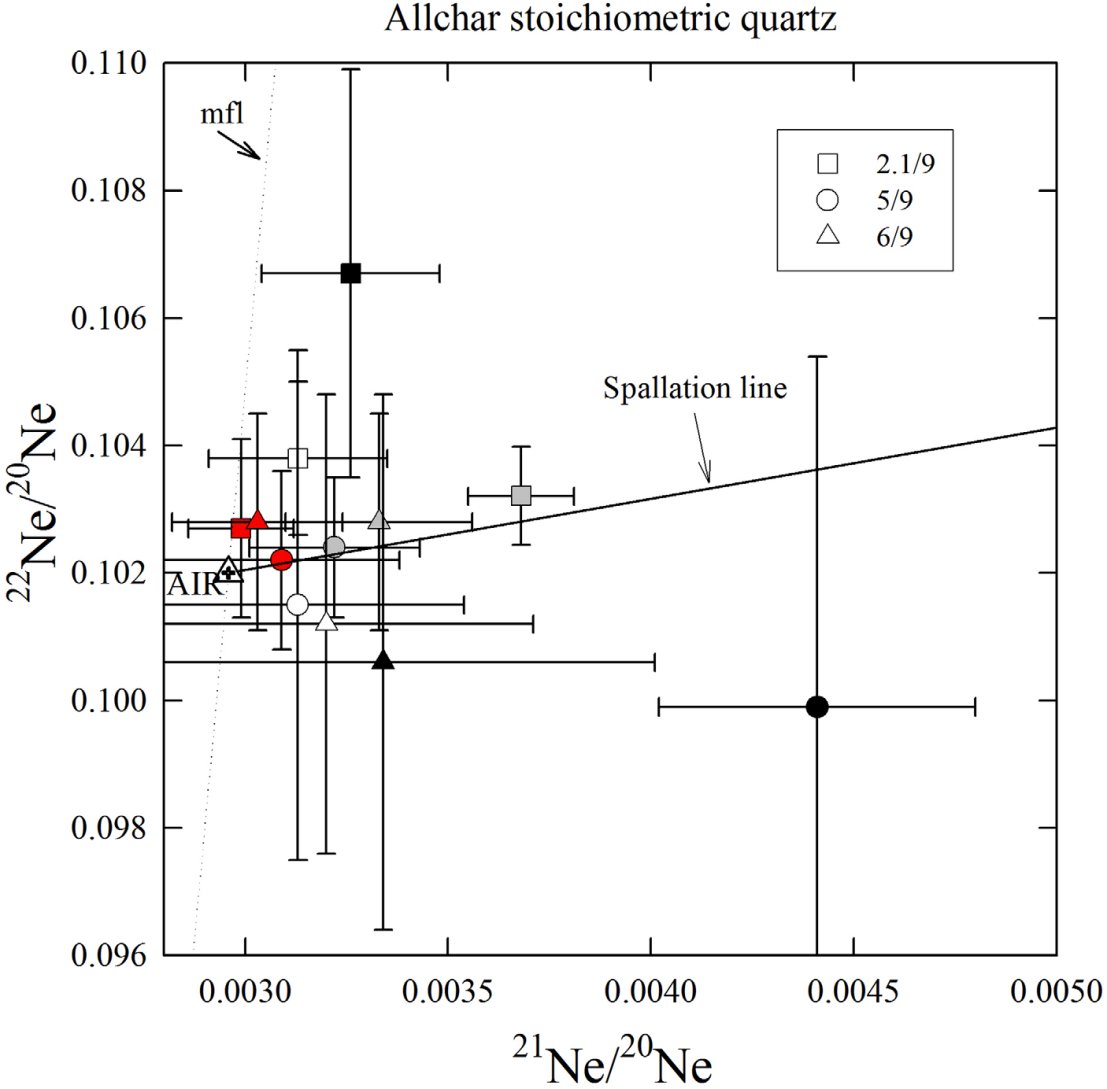
Neon three‐isotope plot showing the stepwise heating extractions of three stoichiometric quartz samples from Allchar. Colors in symbols indicate heating steps: white—400°C, gray—600°C, red—800°C, and black—1200°C. All 400–800°C data are consistent within 2σ error limits with the spallation line [*Niedermann et al*., 1993], i.e., with two‐component mixtures of atmospheric and cosmogenic Ne. Contributions of 1200°C steps, where no cosmogenic Ne is released any more [*Niedermann*, 2002], are small as reflected by large error bars. The mass fractionation line (mfl) is shown for reference. Note that unlike the data in Table 4, those plotted here do not include blank corrections, which would only increase the relative deviations from atmospheric composition as well as the uncertainties without changing the general picture.

**Figure 3:**
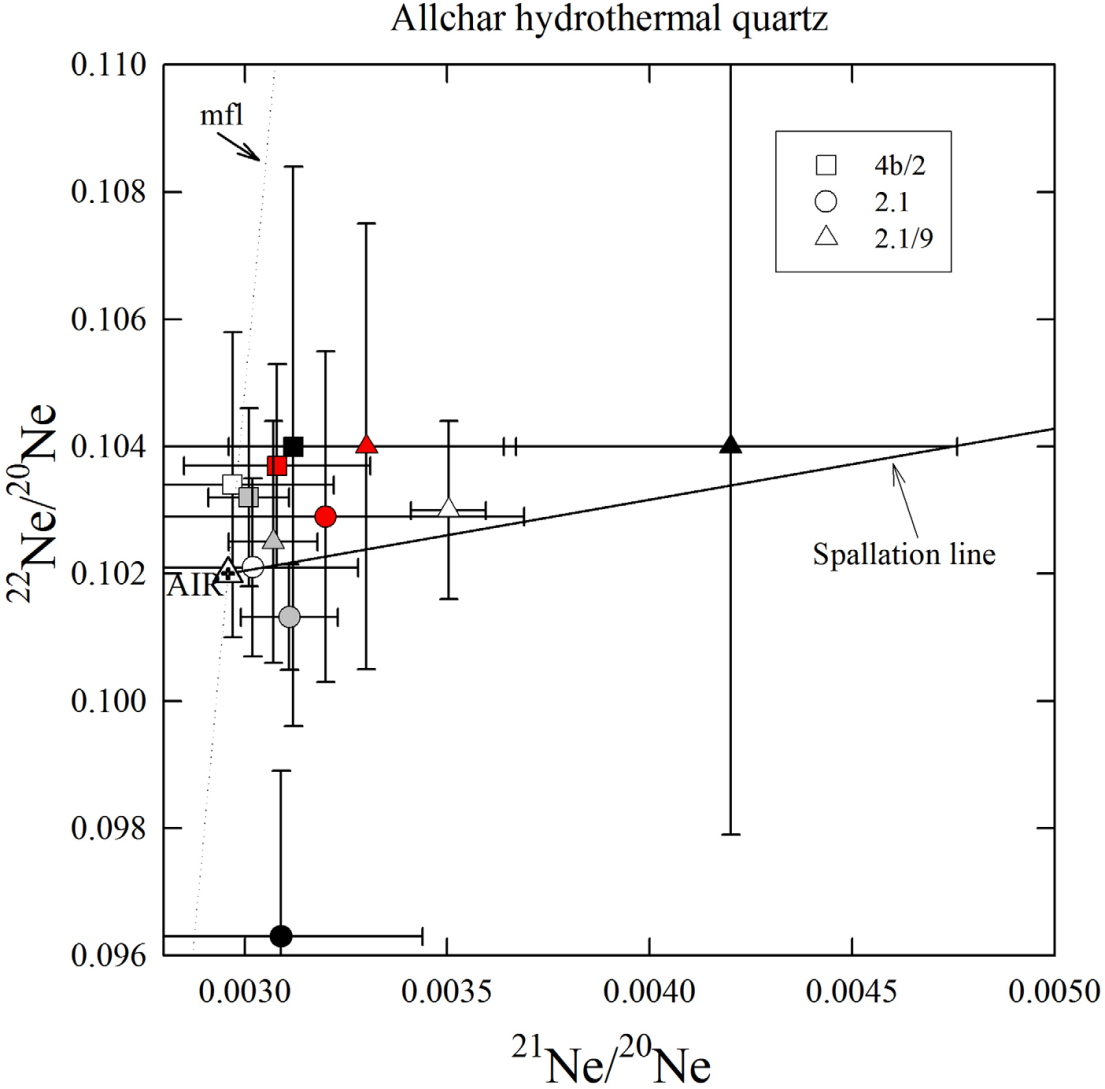
Neon three‐isotope plot showing the stepwise heating extractions of three hydrothermal quartz samples from Allchar. See Figure 2 for explanations.

Results of He and Ne analyses are shown in Tables 4 and 5, respectively. The ^21^Ne/^20^Ne ratios are only slightly above the atmospheric value (0.002959) in most heating steps of all quartz and sanidine samples. When plotted in a three‐isotope diagram (Figures 2 and 3), all 400–800°C quartz data overlap with the “spallation line” [*Niedermann et al*., 1993] within error limits, indicating two‐component mixing between atmospheric and cosmogenic Ne. Above 800°C, no cosmogenic Ne is released from quartz [*Niedermann*, 2002]. Therefore, cosmogenic Ne in the quartz samples was calculated from the excesses over atmospheric composition in the 400–800°C heating steps (Table 5); the small excesses observed in the 1200°C steps are interpreted to be nucleogenic Ne produced by the reaction ^18^O(α,n)^21^Ne, with α particles derived from U or Th decay. In the sanidine sample, the ^21^Ne excess is barely significant at the 1σ level (not significant at the 2σ level), thus the total concentration of cosmogenic ^21^Ne based on the 600°C and 800°C steps [*Kober et al*., 2005] should be considered as an upper limit rather than a robust figure (Table 5). Similarly, cosmogenic ^3^He in the diopside sample can only be reported as an upper limit, assuming that all ^3^He measured in this sample (87.3 ± 3.9 × 10^5^ at/g) is cosmogenic. Prior to the stepwise heating extraction, this sample had been crushed in vacuo in order to characterize the composition of trapped noble gases. However, the He released by this method was very small, <1% of that extracted by heating (Table 4), and showed an imprecise ^3^He/^4^He ratio of (0.18 ± 0.11) × 10^−6^. This indicates that the major ^4^He fraction in this sample is radiogenic and represents a component which resides in the crystal lattice and is thus not released by crushing. This interpretation is further supported by the isotopic composition of Ne (Table 4) and the ratio of excess ^21^Ne to ^4^He of (4.22 ± 0.19) × 10^−8^. The latter value is almost identical to the production ratio of nucleogenic ^21^Ne to radiogenic ^4^He (4.5 × 10^−8^) [*Yatsevich and Honda*, 1997]. Since the ^3^He/^4^He ratio of radiogenic He is on the order of 10^−8^, while the measured ^3^He/^4^He ratio was about 1.5 × 10^−7^, it is reasonable to assume that the major fraction of ^3^He is cosmogenic. Nevertheless, we can only report cosmogenic ^3^He as an upper limit.

### 3.2 Estimates of the Erosion Rate at the Allchar Deposit

#### 3.2.1 Maximum Erosion Rates Based on Individual Cosmogenic Nuclides

In a first step, we have calculated maximum erosion rates for the 10 sampling locations in Allchar based on individual cosmogenic nuclides. Table 6 presents the values of oblique and vertical erosion rates (see below) estimated by four individual cosmogenic nuclides in various monitoring minerals (total stoichiometric and hydrothermal quartz, dolomite/calcite, sanidine, and diopside).

**Table 4:** Results of Stepwise Heating He and Ne Analyses in Quartz, Sanidine, and Diopside Separates From Allchar^c^

		^4^He	^20^Ne	^3^He/^4^He	^22^Ne/^20^Ne	^21^Ne/^20^Ne
Sample, Location, Weight	T (°C)	10^−8^	10^−12^	10^−6^	10^−2^	10^−2^
5/9 quartz^a^	400	0.00458 ± 0.00028	11.23 ± 0.42	5.2 ± 3.1	10.15 ± 0.20	0.313 ± 0.021
L‐3b/RML	600	0.258 ± 0.007	51.6 ± 1.7	0.18 ± 0.07	10.24 ± 0.06	0.322 ± 0.011
0.70814 g	800	1.040 ± 0.026	16.1 ± 0.6	0.040 ± 0.031	10.22 ± 0.07	0.309 ± 0.015
	1200	0.316 ± 0.008	1.99 ± 0.24	0.13 ± 0.07	9.87 ± 0.43	0.523 ± 0.040
	Total	1.619 ± 0.028	80.9 ± 1.9	0.094 ± 0.028	10.21 ± 0.05	0.323 ± 0.008
2.1/9 quartz^a^	400	0.0201 ± 0.0006	36.5 ± 1.2	1.6 ± 0.8	10.38 ± 0.06	0.313 ± 0.011
L‐4/ADR	600	0.492 ± 0.013	135.1 ± 4.4	0.075 ± 0.039	10.321 ± 0.039	0.368 ± 0.007
0.71248 g	800	0.812 ± 0.021	53.4 ± 1.8	0.07 ± 0.06	10.27 ± 0.07	0.299 ± 0.007
	1200	0.284 ± 0.007	12.91 ± 0.47	0.19 ± 0.13	10.67 ± 0.16	0.326 ± 0.011
	Total	1.608 ± 0.025	237.9 ± 4.9	0.112 ± 0.039	10.338 ± 0.030	0.342 ± 0.005
6/9 quartz^a^	400	0.00717 ± 0.00038	8.53 ± 0.33	200 (+600/–200)	10.12 ± 0.18	0.320 ± 0.026
L‐3a/RML	600	0.235 ± 0.006	41.9 ± 1.4	<1.1	10.28 ± 0.09	0.333 ± 0.012
0.70726 g	800	1.398 ± 0.035	22.3 ± 0.8	0.033 ± 0.022	10.28 ± 0.09	0.303 ± 0.011
	1200	0.519 ± 0.013	4.32 ± 0.28	0.07 (+0.08/–0.07)	10.02 ± 0.27	0.344 ± 0.042
	Total	2.159 ± 0.038	77.1 ± 1.7	0.7 (+2.0/–0.7)	10.25 ± 0.06	0.323 ± 0.008
Sa/08 sanidine	600	0.539 ± 0.014	96.9 ± 3.5	0.12 (+0.16/–0.12)	10.272 ± 0.047	0.308 ± 0.010
L‐1a/R	800	0.0929 ± 0.0026	24.8 ± 1.0	0.38 (+0.41/–0.38)	10.45 ± 0.08	0.307 ± 0.013
0.50028 g	1750	0.0217 ± 0.0010	12.8 ± 0.6	2.8 (+2.9/–2.8)	11.16 ± 0.23	0.321 ± 0.015
	Total	0.654 ± 0.014	134.5 ± 3.7	0.25 ± 0.18	10.389 ± 0.043	0.309 ± 0.008
4b/2 quartz^b^	400	0.1518 ± 0.0047	32.2 ± 1.3	0.43 ± 0.36	10.34 ± 0.12	0.297 ± 0.013
L‐1b/R	600	0.428 ± 0.013	147.9 ± 4.6	0.18 ± 0.14	10.32 ± 0.07	0.301 ± 0.005
0.28214 g	800	0.0667 ± 0.0023	69.2 ± 2.5	0.6 ± 0.6	10.37 ± 0.08	0.308 ± 0.012
	1200	0.0018 (+0.0020/−0.0018)	4.8 ± 1.0	4 (+11/–4)	10.58 ± 0.42	0.33 ± 0.05
	Total	0.648 ± 0.014	254 ± 6	0.29 ± 0.14	10.34 ± 0.05	0.303 ± 0.005
2.1 quartz^b^	400	0.0534 ± 0.0019	59.2 ± 2.0	0.6 (+1.3/–0.6)	10.21 ± 0.07	0.302 ± 0.013
L‐2/RT	600	0.0272 ± 0.0013	84.9 ± 2.7	1.3 ± 1.0	10.132 ± 0.042	0.311 ± 0.006
0.29022 g	800	0.0046 ± 0.0009	13.5 ± 1.1	10 ± 8	10.33 ± 0.18	0.329 ± 0.034
	1200	0.0005 (+0.0009/−0.0005)	1.9 ± 1.1	50 (+90/–50)	8.2 ± 1.2	0.34 ± 0.07
	Total	0.0857 ± 0.0026	159.5 ± 3.7	1.6 ± 1.2	10.155 ± 0.042	0.310 ± 0.007
2.1/9 quartz^b^	400	0.0795 ± 0.0025	48.3 ± 1.6	1.4 (+3.5/–1.4)	10.30 ± 0.07	0.3503 ± 0.0047
L‐4/ADR	600	0.219 ± 0.007	43.5 ± 1.5	0.19 (+0.37/–0.19)	10.25 ± 0.10	0.307 ± 0.006
0.33898 g	800	0.1228 ± 0.0039	1.3 ± 0.8	0.37 ± 0.34	11.1 ± 1.0	0.45 ± 0.12
	1200	0.0150 ± 0.0008	<0.23	1.5 (+1.8/–1.5)	‐	‐
	Total	0.436 ± 0.008	93.1 ± 2.4	0.5 (+0.7/–0.5)	10.29 ± 0.06	0.3315 ± 0.0041
Di/09 diopside	900	204 ± 5	69.9 ± 2.6	0.143 ± 0.006	10.89 ± 0.11	0.346 ± 0.010
L‐7/KR	1750	14.93 ± 0.38	6.8 ± 1.2	0.222 ± 0.037	13.6 ± 0.7	1.13 ± 0.15
0.37090 g						
	Total	219 ± 5	76.7 ± 2.9	0.148 ± 0.006	11.13 ± 0.12	0.416 ± 0.019
0.37852 g	Crushed	1.348 ± 0.034	37.9 ± 1.2	0.18 ± 0.11	10.35 ± 0.12	0.298 ± 0.013

a Total stoichiometric quartz.

b Hydrothermal quartz.

Noble gas concentrations are in units of cm^3^ STP/g, error limits are 1σ.

**Table 5:** ^21^Ne Excesses (Relative to Atmospheric Isotopic Composition, in Units of 10^5^ at/g) as Determined by Stepwise Heating in Total Stoichiometric Quartz (*), Hydrothermal Quartz (**), and Sanidine (Sample Sa/08)^a^

Sample/Temp. (°C)	2.1/9*	5/9*	6/9*	4b/2**	2.1**	2.1/9**	Sa/08
400°C	1.7 ± 1.1	0.52 (+0.61/–0.52)	0.55 (+0.57/–0.55)	0.1 (+1.1/–0.1)	1.0 (+2.1/–1.0)	7.06 ± 0.63	
600°C	26.3 ± 2.4	3.7 ± 1.5	4.2 ± 1.3	2.1 ± 2.0	3.3 ± 1.3	1.32 ± 0.65	3.2 ± 2.6
800°C	0.5 (+1.0/–0.5)	0.57 (+0.61/–0.57)	0.39 (+0.64/–0.39)	2.2 ± 2.1	1.2 (+1.3/–1.2)	0.52 ± 0.26	0.78 (+0.85/–0.78)
1200°C	1.04 ± 0.37	1.22 ± 0.17	0.56 ± 0.49	0.39 (+0.68/–0.39)	0.22 (+0.30/–0.22)	0.76 ± 0.18	0.86^b^ ± 0.50
Total ≤800°C	28.5 (+2.8/–2.7)	4.8 ± 1.7	5.1 (+1.6/–1.5)	4.4 (+3.1/–2.9)	5.5 (+2.8/–2.0)	8.90 ± 0.94	4.0 ± 2.7

a Total cosmogenic^21^Ne concentrations are calculated as the sums of the excesses in the ≤800°C steps. Error limits are 1σ.

b 1750°C step.

**Table 6:** Oblique and Vertical Erosion Rates as Determined by ^26^Al, ^36^ Cl, ^21^Ne, ^3^He at Different Locations in Allchar^a^

Location	Sample Name	^26^Al (10^5^ at/g SiO_2_)	Erosion rate (m/Ma)	Vert. erosion rate (m/Ma)	^36^Cl (10^5^ at/g)	Erosion rate (m/Ma)	Vert. erosion rate (m/Ma)	^21^Ne (10^5^ at/g SiO_2_)	Erosion rate (m/Ma)	Vert. erosion rate (m/Ma)	^3^He (10^5^ at/g diops.)	Erosion rate (m/Ma)	Vert. erosion rate (m/Ma)
L‐7/KR	Di/09										≤95	≥15	≥15
L‐8 CD	Do 8/13				2.16	365	387						
					(0.30)	(+86/−75)	(+92/−80)						
L‐1a/R	Sa/08							4.0^b^	59	69			
								(2.7)	(+123/−24)	(+145/−28)			
L‐1b/R	4b/2	1.2	331	400				4.4	42	51			
		(1.0)	(+1700/−150)	(+2000/−180)				(+3.1/−2.9)	(+81/−17)	(+99/−21)			
L‐1c/R	Do 50L/13				3.70	210	300						
					(0.31)	(+55/−52)	(+78/−74)						
L‐2/RT	2.1							5.5	29	41			
								(+2.8/−2.0)	(+17/−10)	(+24/−14)			
L‐3a/RML	3.2/2;	6.7	63	67				5.1	37	39			
	3.2/2^c^;6; 6/2‐1;6/2‐2; 6/9	(1.2)	(+15/−11)	(+16/−12)				(+1.6/−1.5)	(+15/−9)	(+16.0/−9.0)			
L‐3b/RML	3.1/2;	4.8	85	94				4.8	37	41			
	3.1/2^c^;5;5/9	(0.8)	(+19/−14)	(+21/−16)				(1.7)	(+20/−10)	(+22/−11)			
L‐3c/RML	Do 1/13				5.23	135	164						
					(0.44)	(+41/−36)	(+50/−44)						
L‐4/ADR	1.3/2;	1.3	332	345				8.9	21.2	22			
		(1.1)	(+1800/−160)	(+1860/−165)				(0.9)	(+2.5/−2.0)	(+3/−2)			

a Average values are shown where several samples were analyzed for the same nuclide.

b 10^5^ at/g sanidine.

c Duplicate measurements.

Production rates (resulting from spallation, slow muon capture, and fast muon‐induced reactions) were calculated on the basis of geographical position of the sample locations (Table 1) using scaling factors according to *Dunai* [2001] for ^26^Al and ^36^Cl and *Dunai* [2000] for ^3^He and ^21^Ne. We assumed sea level and high latitude (SLHL) production rates for spallation of 29.9 at/g SiO_2_/a for ^26^Al [*Balco et al*., 2008], 48.8 at/g Ca/a for ^36^Cl [*Stone et al*., 1996], 19.86 at/g SiO_2_/a for ^21^Ne [*Goethals et al*., 2009], and 124 at/g diopside/a for ^3^He [*Goehring et al*., 2010] (combined olivine and pyroxene production rate). The ^21^Ne production rate in sanidine was assumed to be ∼1.5 times that in quartz [*Kober et al*., 2005]. This rough value is accurate enough considering the large uncertainty of the cosmogenic ^21^Ne concentration in our sanidine sample Sa/08 (Table 5). The contribution of muon‐induced reactions to the total production was assumed to be zero for ^3^He and ^21^Ne [*Schoenbohm et al*., 2004].

Two additional corrections have been applied to the acquired data for the calculation of erosion rates on sloped surfaces. First, we considered the dip angles of the sampled surfaces, i.e., we calculated shielding factors for sloped surfaces as given in equations (10) and (11) of *Niedermann* [2002] to account for the shielded fraction of cosmic rays. We then calculated erosion rates ε using the relation

(2) $$C=\frac{P_{sp}\text{exp}(-\frac{\rho h}{\Lambda_{sp}})}{\lambda +\frac{\rho \varepsilon }{\Lambda_{sp}}}+\frac{P_{m}\text{exp}(-\frac{\rho h}{\Lambda_{m}})}{\lambda +\frac{\rho \varepsilon }{\Lambda_{m}}}+\frac{P_{f}\text{exp}(-\frac{\rho h}{\Lambda_{f}})}{\lambda +\frac{\rho \varepsilon }{\Lambda_{f}}}$$

where Λ_sp_ = 160 g/cm^2^, Λ_m_ = 1510 g/cm^2^, and Λ_f_ = 4320 g/cm^2^ are the attenuation lengths for spallation, muon capture, and production induced by fast muons, respectively, ρ = 2.71 g/cm^3^ is adopted for the density of the studied rocks, P_sp_, P_m_, and P_f_ are the (local) production rates on the sloped surface (see above) through spallation, muon capture, and production by fast muons, respectively, C is the cosmogenic nuclide concentration, h is the present depth of the samples, and λ is the decay constant of radionuclides (λ = 0 for ^3^He and ^21^Ne). Second, we applied an additional correction to the erosion rates according to equation (1) of *Hermanns et al*. [2004], which takes into account the reduced attenuation length beneath sloped surfaces and is based on earlier work of *Dunne et al*. [1999].

The resulting values determined in this way for ^26^Al in quartz agreed very well with those obtained by the CRONUS Earth online calculator (http://hess.ess.washington.edu/) [*Xiuzeng et al*., 2007; *Balco et al*., 2008] when the above corrections are included; the latter values are reported in Table 6. Finally, vertical erosion rates, i.e., downwearing rates of the overall horizontal landscape, were estimated by dividing those acting on inclined surfaces by the cosine of the dip angle (Table 6). We did not include any corrections for vegetation or snow cover, because they are difficult to assess over geological timescales, but most probably insignificant. For example, *Plug et al*. [2007] estimate a 2.3% effect for temperate (Acadian) forest.

#### 3.2.2 ^26^Al‐^21^Ne Combination

**Figure 4:**
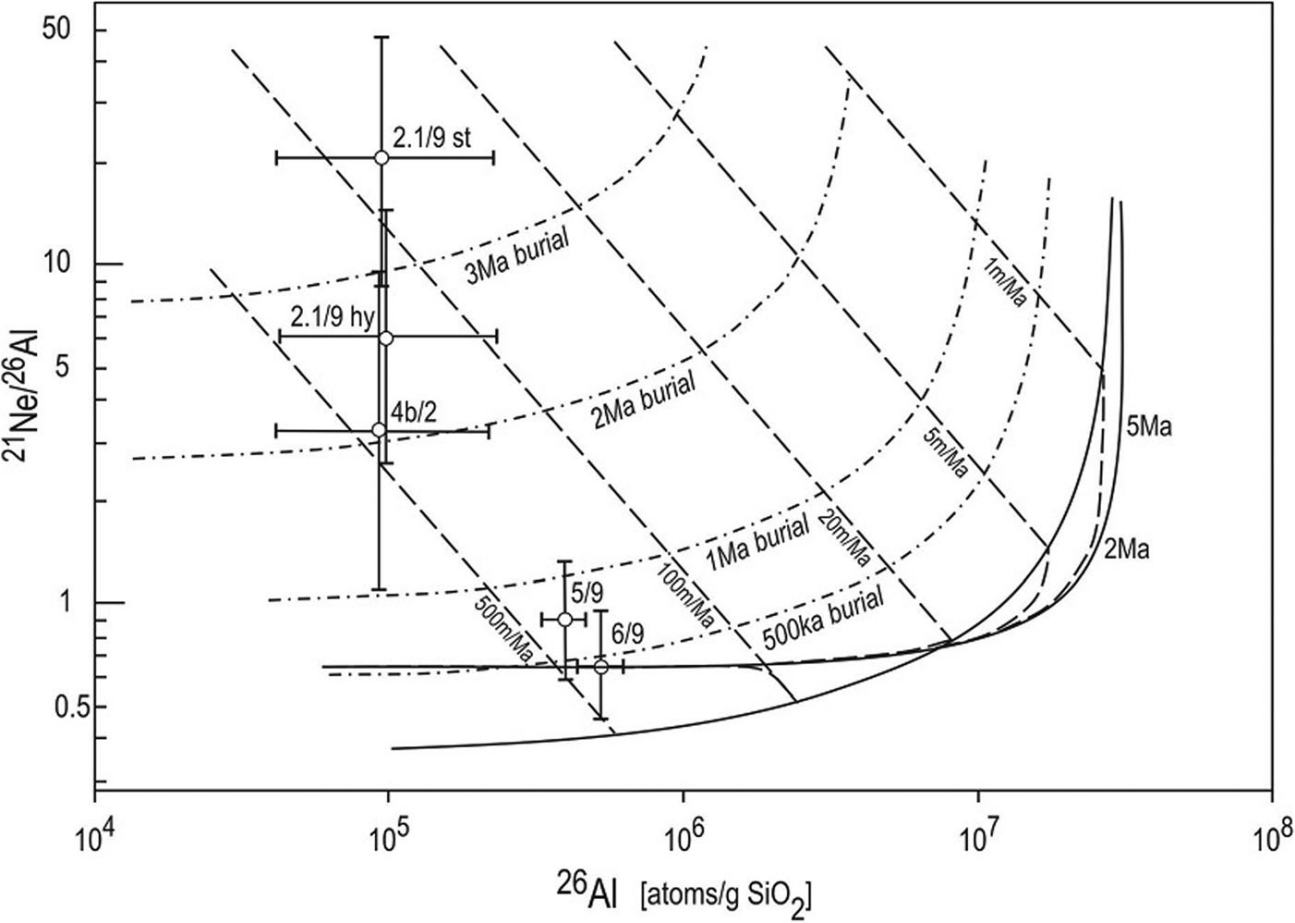
Plot of ^21^Ne/^26^Al versus ^26^Al concentrations (scaled to sea level and high latitude) showing data from four Allchar locations. Most data lie in the burial field, indicating a combination of cosmic‐ray exposure and erosion with intermittent burial periods. See text for discussion. Graph was drawn in CosmoCalc version 1.8 [*Vermeesch*, 2007].

Figure 4 shows the “erosion island plot” for ^21^Ne/^26^Al versus ^26^Al. All data either overlap with the steady state erosion island or lie above it in the complex exposure history field. Therefore, the combined ^21^Ne and ^26^Al data can be explained by scenarios involving one or more periods of exposure (with or without erosion) and one or more intermittent periods of burial. In case of samples 5/9 and 6/9 from locations L‐3b/RML and L‐3a/RML, respectively, the data would even allow for a simple exposure history with no erosion and no burial. However, this is not reasonable in the geological context (see the next section). More likely, the positions of the data points indicate a combination of erosion rates in the range from about 100 to 500 m/Ma and burial periods lasting for ∼0.5–3.5 Ma.

## 4 Discussion

### 4.1 Geological Considerations

Geological data indicate that during and particularly after volcanic activity, the rocks in Allchar underwent erosion and redeposition in adjacent terrain depressions. Because parts of the terrain, which were sampled for this cosmogenic nuclide study, have likely undergone multiphase periods of burial by variably thick volcaniclastic material, their exposure time must have been different as well.

The following geological and geochronological information should be taken into consideration before interpreting the erosion rates in the area of Allchar:

1. The time span of volcanic activity was between 6.5 and 1.7 Ma [*Boev*, 1988].
2. Alteration of the wall‐rocks in the surroundings of the ore bodies Crven Dol and Centralni Deo, as well as formation of thallium mineralization, commenced at 4.3 Ma.
3. The maximal thickness of volcaniclastic material may have been up to several hundreds of meters.
4. After the termination of volcanic activity, the volcaniclastic material was redeposited and accumulated in the low level surroundings (depressions and river valleys).
5. There is no solid evidence about the existence of glaciers in the area of Allchar.
6. The relevant erosion period lasted from the time of formation of the ore bodies until present day, and was constantly influenced by exogenous processes.

All the above information about the Pliocene history of Allchar suggests that this area must not be considered as a stationary rock complex. Rather than that its surface was constantly changing in the relevant period of 4.3 Ma.

### 4.2 Episodes of Exposure and Burial

Any stable geological surface that is exposed to cosmic rays accumulates cosmogenic nuclides. In principle, two major cases can be elaborated, namely, eroded and noneroded surfaces. In the Allchar case, we consider an eroded surface at various locations that are characterized by variable lithology. We may assume that for a comparably short time τ_ε_ (up to a few thousand years), the erosion rate ε was constant [e.g., *Lal*, 1991; *Niedermann*, 2002]. If we calculate apparent exposure ages τ_ε_ = C_21_/P_21_ (where C_21_ is the ^21^Ne concentration and P_21_ its production rate) for different Allchar locations on the basis of ^21^Ne concentrations, postulating that during the last 100 ka the area of Allchar was neither covered by glaciers nor influenced by any volcanic activity (see section 4.1), then 9 ka ≤ τ_ε_ ≤ 93 ka. The high value of 93 ka is an exception; most values being around 20 ka or less. For surfaces undergoing erosion, τ_ε_ corresponds to the characteristic erosion timescale, i.e., the time it takes to erode the thickness of rock equivalent to one attenuation length. This is the timescale over which our measurements integrate. As noted earlier, the “individual‐nuclide” erosion rate values given in Table 6 must be regarded as upper limits, because any deviation from the assumed “simple exposure history” will cause an overestimate of the mean erosion rate over the relevant time period. However, in exposure scenarios involving burial the true erosion rate during sufficiently short periods when the surface was not covered may nevertheless have been higher than the calculated “maximum erosion rate” for the whole period, because burial (or sedimentation) is equivalent to “negative erosion” and thus reduces the mean. As it is clearly not possible to apply a linear erosion model (i.e., a steady state irradiation history) for the whole period between the Pliocene (5.33–2.58 Ma) and Holocene (the last 11.5 ka) at Allchar, more complex scenarios of the exposure to cosmic rays must be considered.

Given that volcanic activity in the Allchar region lasted from 6.5 to ∼1.7 Ma, it can be supposed that it could have caused fast deposition of primary pyroclastic or volcaniclastic (redeposited) material. Such rapid burial of the surface rocks could have stopped the neutron‐controlled production of cosmogenic nuclides for as long as 4 Ma. This time period corresponds to almost six half‐lives of ^26^Al. In other words, during the time of burial, the ratio ^21^Ne/^26^Al would have increased ∼60 times. However, in the last Pleistocene/Holocene epochs, from 200 to 300 ka to present, new phases of erosion and redeposition into adjacent depressions and river valleys may have occurred. During that time surface rocks would have been again exposed to cosmic radiation and this would have decreased the ^21^Ne/^26^Al ratio again. The erosion island plot for ^21^Ne and ^26^Al (Figure 4) is in agreement with such a supposed geological history of Allchar. The high ^21^Ne/^26^Al ratios imply a relatively long time of sedimentation (high duration of burial), some 2–3 Ma at the locations L‐1b/R and L‐4/ADR and probably several hundreds of ka at L‐3a/RML and L‐3b/RML. A smaller burial duration at the latter locations is reasonable because they are closer to the mountains and may thus have been uncovered earlier.

In a model scenario involving a single burial episode, the stable cosmogenic nuclides ^21^Ne and ^3^He from the analyzed samples originate from two periods: a preburial time, which lasted from the quartz crystallization to the moment when accumulation of volcaniclastic material occurred, and a postburial time, which started when the cover was removed and lasted until present time. In contrast to ^21^Ne and ^3^He, the major fractions of ^26^Al and ^36^Cl would have been produced during the last period of exposure, because ^26^Al and ^36^Cl that was present before burial decayed almost completely during the long duration of burial. This is why the ratio ^21^Ne/^26^Al is higher than in case of a “simple exposure history,” i.e., when assuming a steady state erosion model. Therefore, ^26^Al and ^36^Cl may yield reasonable estimates of the erosion rate prevailing in the postburial episode, i.e., over the last few tens of ka. Of course, the data do not allow us to infer an exposure history involving just one burial period. There may have been several successions of exposure and burial, but nevertheless, it is likely that the ^26^Al and ^36^Cl concentrations are most closely representing the erosion rate during the most recent exposure period.

### 4.3 Vertical Erosion Rates in Allchar Based on TCN

As already mentioned above, the main purpose of this study is to determine vertical erosion rates in a 1.5 km long and 0.3 km wide, north‐south elongated area of the Allchar deposit (see Figure 1). Because this area underwent a complex geological history, we applied different nuclides (both radioactive—^26^Al and ^36^Cl and stable—^3^He and ^21^Ne) on different monitoring minerals (magmatic and hydrothermal quartz, dolomite/calcite, diopside, and sanidine) in order to acquire relevant information about prevailing erosion rates. The results are summarized in Table 6, where vertical erosion rate estimates are given in m/Ma for all the applied cosmogenic nuclides.

The results show accordance in the values obtained by applying ^26^Al, ^36^Cl, ^3^He, and ^21^Ne. Hence, at sample locations L‐2/RT, L‐3a/RML, L‐3b/RML, and L‐4/ADR (except for ^26^Al) the obtained erosion rate values vary between several tens of m/Ma to hundreds of m/Ma. These values are similar to those from earlier AMS measurements, albeit on a considerably smaller number of samples. The erosion rates determined earlier based on ^26^Al at the locations L‐3a/RML and L‐3b/RML are 49 and 59 m/Ma, respectively, whereas based on ^53^Mn a rate of ≥40 m/Ma at the location closest to L‐1a/R was estimated [*Pavićević et al*., 2010]. However, the ^26^Al data from the localities L‐1b/R, L‐1c/R, L‐4/ADR, and L‐8/CD imply remarkably higher erosion rate values, which may potentially be explained by abrupt exhumations (sudden exposure to cosmic radiation), which must have occurred in the recent geological past in this part of Allchar. In spite of the mentioned differences, it can be postulated that a conservative lower limit to the prevailing erosion rates in the Allchar area is between 50 and 100 m/Ma. This range enables us to estimate vertical erosion rate values for the immediate locations of the ore bodies of Crven Dol and Centralni Deo.

### 4.4 Paleo‐Depth Estimates of the Crven Dol and Centralni Deo Ore Bodies

A principle factor for the success of the LOREX Project is relevant information about the erosion rate of the rock in the Allchar area for the last 4.3 Ma, which is the geological age of the Allchar deposit. Namely, in case of very low erosion rates, a small ratio of the experimentally determined total concentration of ^205^Pb in lorandite, i.e., (N^205^Pb)_exp_ in equation (1), and the concentration of ^205^Pb produced by cosmic irradiation and other background processes, i.e., (N^205^Pb)_B_ in equation (1), could potentially harm the project feasibility [*Jelenković and Pavićević*, 1990]. The ratio (N^205^Pb)_exp_/(N^205^Pb)_B_ in lorandite is predominantly depth‐dependent, which means that it depends on the average depth of the lorandite from the time when the mineralization precipitated to the present day [*Pavićević et al*., 2010]:

(3) $$d_{p}=d_{today}+\frac{1}{2}\varepsilon \cdot T$$

where *d_today_* is the present‐day depth of the lorandite ore and *ε* is the average erosion rate for the time period *T* = 4.3 Ma. Although this is a simplified relation, neither taking into account the complex depth dependence of cosmogenic nuclide production by spallation and muon interactions nor the very probable variations of the erosion rate in the past, it is sufficient for a first‐order estimate.

*Pavićević et al*. [2012] reported an estimate of the present amount of ^205^Pb produced in lorandite due to solar neutrinos and background reactions in the last 4.3 Ma as a function of the paleo‐depth of the lorandite [*Pavićević et al*., 2012, Figure 2]. Furthermore, it is straightforward to estimate the present‐day depth of the lorandite, taking into account the available information about mining horizons and geological boreholes in the ore bodies Crven Dol and Centralni Deo. However, assessing the depth of the eroded layer (equation (3)) by applying TCN is a delicate task, especially bearing in mind that the Allchar area underwent a complex geological evolution (see sections 4.1 and 4.2).

As argued above, the erosion rate values that we obtained by applying the radioactive nuclides ^26^Al and ^36^Cl (Table 6) are valid only for the last geological past—essentially the Holocene. On the other hand, the concentrations of cosmogenic ^21^Ne and ^3^He (Table 6) reflect a combination of erosion and burial during the total geological history of the Allchar area, from the Lower Pliocene to the present day. Therefore, the estimates of erosion rate obtained from the stable nuclides ^21^Ne and ^3^He can be considered as closer to “long‐term average values” than those that are calculated on the basis of the radioactive nuclides ^26^Al and ^36^Cl. Consequently, the erosion rate estimates based on stable nuclides and those obtained by radioactive nuclides are considered minimal and maximal erosion rates in the Allchar area, respectively.

On the basis of the erosion rate values reported in Table 6, equation (3) and the values of d_today_ (28 m for the ore body Crven Dol 1, 103 m for the shallower part of the ore body Centralni Deo 3, and 140 m for the deeper part of the ore body Centralni Deo 4) [*Pavićević et al*., 2012, Table 1], we estimate a maximum paleo‐depth value for the ore bodyCrvenDol(d_1_) as well as minimum and maximum values for the ore bodyCentralniDeo (d_3_ and d_4_) are as follows:

$$\begin{array}{cc} d_{\text{max}}^{1\text{Crv}.D}=\;860m^{\;+200m}{/}_{-180m} & \\ d_{\text{min}}^{3\text{Cen}.D}=\;250m^{\;+310m}{/}_{-60m}; & d_{\text{max}}^{3\text{Cen}.D}=750m^{\;+170m}{/}_{-160m}\\ d_{\text{min}}^{4\text{Cen}.D}=\;290m^{\;+310m}{/}_{-60m}; & d_{\text{max}}^{4\text{Cen}.D}=\;790m^{\;+170m}{/}_{-160m} \end{array}$$

These values should be multiplied by a factor of 2.71 to obtain paleo‐depths in units of meter water equivalent (mwe).

At the Crven Dol locality, we did not have samples in which the stable nuclides ^3^He or ^21^Ne could be measured, and this is the reason why we do not have a lower limit estimate of the paleo‐depth there. In spite of this, these data provide a reasonable basis for estimating the pp‐neutrino and fast muon contributions to^205^Pb production.

## 5 Conclusions

Based on the combined analysis of ^26^Al and ^21^Ne in a subset of our samples (Figure 4), it can be concluded that the Allchar area underwent a complicated geological history and that cosmic irradiation of the surface rocks reflects erosion rates ranging from ∼100 to 500 m/Ma and burial ages between ∼500 ka and 3 Ma.

By estimating the maximum erosion rates at 10 different localities of Allchar (within an area of around 0.3 km × 1.5 km), applying two radioactive (^26^Al and ^36^Cl) and two stable (^3^He and ^21^Ne) nuclides in quartz, calcite/dolomite, sanidine, and diopside as monitoring minerals, we established that 85% of the values range from several tens of m/Ma to 350 m/Ma, depending on the location.

The obtained range of erosion rates based on stable and radioactive nuclides enables us to estimate reasonable limits for the vertical erosion rate values at the locations of the main lorandite ore bodies Crven Dol and Centralni Deo. On this basis, we estimate that the minimum and maximum paleo‐depths of the ore body Centralni Deo (averaged from 4.3 Ma to present) are 250–290 and 750–790 m, respectively. In relation with this, we further estimate a maximum paleo‐depth for the ore body Crven Dol (over the same geological age) of around 860 m.

These values allow us to determine the “upper and lower limit” of the contributions of ^205^Pb (number of atoms ^205^Pb/g of lorandite), which have been caused by interaction of fast cosmic‐ray muons over 4.3 Ma in Allchar lorandite. As a result, the ratio of signal (contribution of ^205^Pb by pp‐neutrinos) to background (contribution of ^205^Pb by fast muons) is in the interval from 1:1 to 4:1, which implies a 1σ error of the mean neutrino flux δ = 20% to 30%, lower than the value estimated in *Pavićević et al*.[2012].
